# Comprehensive single-cell sequencing reveals the tumor microenvironment and tumor-specific characteristics in trachea squamous cell carcinoma

**DOI:** 10.3389/fonc.2025.1575647

**Published:** 2025-08-12

**Authors:** Hongwu Wang, Hongli Li, Heng Zou, Han Meng, Yan Liu, Chengjun Ban, Weixia Yu, Miao Cheng, Jun Teng

**Affiliations:** Dongzhimen Hospital, Beijing University of Chinese Medicine, Beijing, China

**Keywords:** single-cell sequencing, tumor microenvironment, cell-cell interaction, chemotherapy, trachea squamous cell carcinoma

## Abstract

Trachea squamous cell carcinoma (TSCC) is a subtype of lung cancer. A thorough investigation of the tumor microenvironment of TSCC is crucial for the development of cancer therapeutics and predicting clinical responses. In this study, we utilized single-cell RNA sequencing to analyze seven TSCC samples (including five malignant and two non-malignant samples) and obtained 70,682 high-quality cells. Based on the expression levels of marker genes, we identified 7 major cell types within the samples. By comparing malignant samples that received chemotherapy with those that did not, we identified critical transcriptional regulators responsible for T cell state transition in response to chemotherapy. Additionally, we found specific transcriptional regulators and differentially expressed genes between malignant and non-malignant groups. We identified more particularly abundant specifical intercellular communication in the malignant sample group and that may significantly influence the progression and spread of cancerous cells. Overall, our study provides the first single-cell atlas that comprehensively explains TSCC development and chemotherapy effects, thereby laying a new molecular foundation for therapeutic research in TSCC.

## Introduction

Lung cancer, including small cell lung cancer and non-small cell lung cancer, has one of the highest incidences and mortality rates of any malignant tumor in our country and around the world (1). Trachea squamous cell carcinoma (TSCC), an important histopathological subtype of non-small cell lung cancer, originates in the bronchial epithelium, undergoes squamous metaplasia, followed by dysplasia and *in situ* carcinoma, ultimately leading to invasive carcinoma (2, 3).

As a result, there are currently no efficient and reliable drug therapies available specifically targeting TSCC, and the 5-year survival rates of TSCC patients are lower compared to other lung cancer subtypes, resulting in a significant number of patient deaths worldwide (4, 5). Late-stage lung cancer is notoriously challenging to treat, and cytotoxic drugs, which are the standard therapy for treating late-stage TSCC, often have poor therapeutic effects. Most patients do not respond to first-line and second-line pharmacological treatments (6).

Cancer cells, infiltrating immune cells, and cancer-associated fibroblasts make up the tumor ecosystem and manipulate signaling molecules to regulate tumor progression and response to therapy (6, 7). The interactions between these cells allow invading cancer cells to overcome constraints imposed by stromal substances, ultimately leading to malignant lung tumors. Malignant lung tumors can then disseminate from the primary site to distant locations, with cancer metastasis responsible for over 90% of cancer fatalities (8, 9). In contrast, benign lung tumors do not metastasize to other regions of the body (10).

Previous studies have presented a substantially comprehensive characterization of the cell types present in advanced NSCLC, including cancer cells, immune cells, and stromal cells, using scRNA-seq analysis (7–11). In order to gain a better understanding of the dynamics and molecular characteristics of the immune landscape in NSCLC, single-cell immune landscapes at high resolution have been depicted (12, 13). However, the cell type landscape and biological differences between benign and malignant TSCC remain largely uncharted.

To fill this gap, we conducted single-cell RNA sequencing on seven patients, including five malignant and two non-malignant samples. In-depth comparative analyses of stromal and immune cells derived from the malignant and non-malignant TSCC samples revealed molecular heterogeneity within the tumor microenvironment and the underlying mechanisms of tumor progression from cell-cell interaction at the molecular level.

## Materials and methods

### Patients

This study was approved by the Ethics Committee of Dongzhimen Hospital, Beijing University of Traditional Chinese Medicine (2021DZMEC-015-02), and all patients signed informed consent forms for participation. This study was conducted in line with the principles of the Declaration of Helsinki. A total of seven patients, comprising five patients with squamous carcinoma and two patients with benign granuloma post-tracheotomy, underwent interventional bronchoscopy at the Respiratory Department of Dongzhimen Hospital, Beijing University of Chinese Medicine, and relevant tissues were obtained during the procedure. The squamous lung cancer patients were all male, consisting of two patients with early-stage and three patients with advanced squamous lung cancer (of which one had not received treatment during the early stage). The two patients with benign granuloma of the airway were one male and one female.

### Tissue dissociation and cDNA synthesis

Airway tissue specimens were excised during bronchoscopy and immediately placed in pre-cooled MACS tissue storage solution (Miltenyi Biotech, Germany) before being transported at 4°C. Each sample, which consisted of 1g of tissue, was then dissociated using a scalpel and the Lung Dissociation Kit (Miltenyi Biotech, Germany) in accordance with the manufacturer’s instructions. The subsequent single cell suspension was filtered through sterile 70μm and 40μm cell filters. Using the Single Cell B Chip Kit (10x Genomics, 1000074), we generated single-cell gel beads in emulsion according to the manufacturer’s instructions. Approximately 6,000 cells were added to each channel, with an estimated target cell recovery of approximately 3,000 cells. In individual GEMs, the released RNA of captured cells was barcoded using reverse transcription. A S1000TM Touch Thermal Cycler (Bio Rad) was used to perform reverse transcription at 53°C for 45 minutes, followed by 85°C for 5 minutes, and held at 4°C. The cDNA was generated, amplified, and then assessed for quality by Capital Biotechnology, Beijing, using an Agilent 4200.

### Single cell RNA-Seq library preparation

Single-cell RNA-seq libraries were created using the Single Cell 3’ Library and Gel Bead Kit V3.1, according to the manufacturer’s instructions. Finally, Lastly, Illumina Novaseq 6000 sequencers were used to sequence the libraries with a depth of at least 100,000 reads per cell using PE150 (pair-end 150 base pair) readings, which was performed by Capital Biotechnology, Beijing. A feature-barcode matrix was produced using the Cell Ranger software’s count module after alignment and UMI counting.

### Process of single-cell data and annotate major cell lineages.

For each scRNA-seq sample, raw gene expression matrices were generated using Cell Ranger (version 1.3.1). Using the Seurat package(version 4.1.0), all malignant and non-malignant gene expression matrices were aggregated and converted into Seurat objects. To ensure data quality, cells with >500 or <8000 expressed genes and >15% mitochondrial counts were removed from the analysis. Using Seurat’s ScaleData function, the remaining cells’ gene expression matrices were normalized by total and mitochondrial read counts. In addition, 3,000 genes differentially expressed, and 30 principal components were used to reduce the dimensions, while batch effects among each sample were eliminated using the Harmony package. For each cluster, we used the FindAllMarkers() function in Seurat (v4.1.0) with parameters: logfc.threshold = 0.25 min.pct = 0.1, p_val_adj < 0.01.Top marker genes were ranked by average log2 fold change and percentage of cells expressing the gene. We choose genes ranked top 20 in each cluster as marker genes. Using literature-supported marker genes, major cell clusters observed in the two-dimensional UMAP representation were annotated to known cell types according to top 20 marker genes.

### Trajectory analysis

Trajectory analysis was conducted separately for CD4^+^ Treg cells, CD4^+^ Tconv cells, and Neu cells using Monocle2. The normalized count matrix was subjected to dimensionality reduction using the DDRTree algorithm with top 30 principal components as input. The pseudotime value was used to order the cells after dimensional reduction was performed by orderCells (). The cell trajectory was then generated from the reduced dimension space and was visualized using plot_cell_trajectory ().

### Cell–cell communication analysis

Cellchat (https://github.com/sqjin/CellChat) was utilized to compute the cell-cell interaction within the malignant and non-malignant groups, respectively, based on the cluster annotation and counts from our single-cell transcriptomics data. We used the default ligand-receptor pair information, as well as gene expression and prior knowledge of how signaling information interacts with their cofactors. We illustrated the cell-cell communication in each group using a circle plot and a chord diagram.

### Regulon analysis

The standard SCENIC procedures were conducted to analyze the cell subpopulation specifical activated regulons as described previously (14). Python package pySCENIC (version 0.9.9) was used to identify expression modules between TF and potential target genes using co-expression of TF genes with other genes, construct regulons and calculate the activity of regulons score. A regulon is defined as an association between a transcription factor and its direct target gene.

### Different expression analysis

Within the three cell types, we used the FindMarkers function within Seurat to identify genes with differential expression between malignant and non-malignant groups (i.e., fibroblast, neutrophil, and macrophage). This involved comparing the studied subcluster cells of the two groups, and marker genes of the subcluster were defined as those with an average expression >1 fold higher, and p-value adjust <0.01 in the studied subcluster than compared subclusters in the other groups. Additionally, significant genes were separated into two groups based on their mean expression in the studied subcluster, compared to those in other subclusters.

### Gene ontology and KEGG analysis

We conducted GO and KEGG enrichment analysis for the selected upregulated genes using the R package “clusterprofile” (15), which is a software integrates genomic, chemical, and phylogenetic data into a comprehensive database. The KEGG pathway enrichment and enriched gene GO terms were defined based on a p-adjust <0.05 threshold for significant enrichment.

## Results

**Figure 1:**
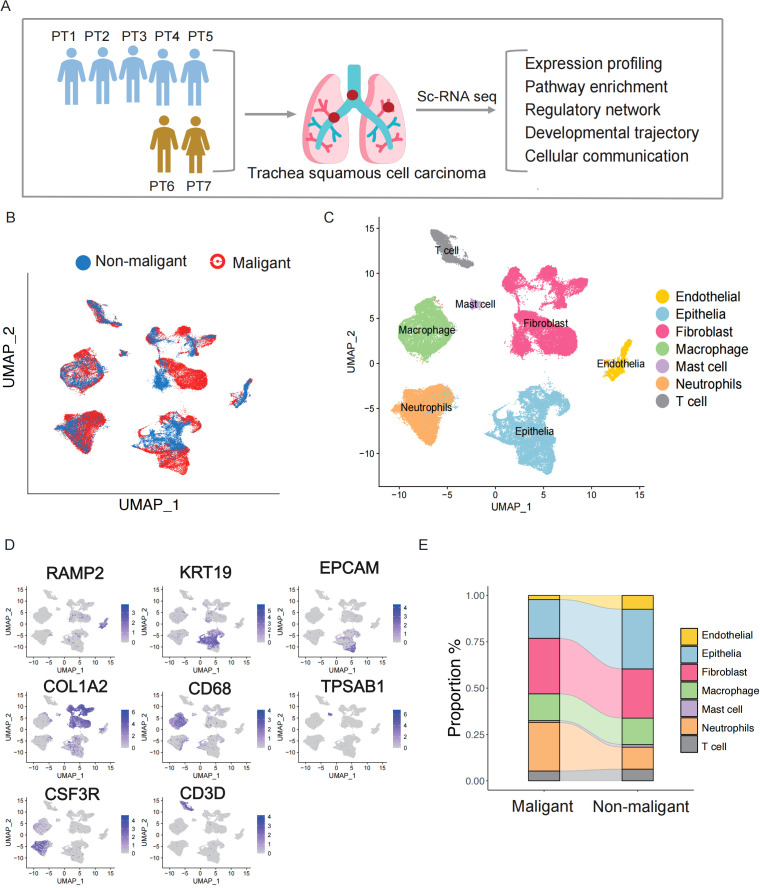
Identification of stromal cells in the TSCC microenvironment via single-cell RNA sequencing. **(A)** Graphical workflow of the experimental design. **(B)** UMAP plot of 66,627 cells and colored by sample type. **(C)** The components in the NPC and NLH microenvironment, according to cell types. **(D)** The expression of marker genes for the identified cell type. **(E)** Stacked bar graph showing the cell fraction of non-malignant samples and malignant samples.

We utilized scRNA-seq to profile seven tissue samples, including five from patients diagnosed with malignant TSCC and two from those diagnosed with non-malignant TSCC. Fresh tissue samples were collected via interventional bronchoscopy, and a customized workflow was used to isolate fresh living single cells primarily from the preprocessed tissue samples. Subsequently, scRNA-seq data analysis was performed to investigate the cell type landscape between malignant and non-malignant samples. The clinical information of these samples is presented in **Supplementary Table S1**, with patient ages ranging from 46 to 71 years. Of the five patients diagnosed with malignant TSCC, three had received chemotherapy treatment. The seven samples were divided into malignant (PT1, PT2, PT3, PT4, PT5) and non-malignant (PT6, PT7) groups (**Figure 1A**).

After mapping the human genome and quality filtering, we obtained 70,682 single cells, of which 45,127 (63.8%) originated from the five malignant samples and 25,413 (36.1%) from the two non-malignant samples, with an average of 2,325 median genes and 8,821 median reads per cell (**Figure 1B**, **Supplementary Figure S1A**). An analysis of downstream processes was performed using Seurat R (version 4.0) (16). Using principal component analysis, dimensionality was reduced, and individual cells were grouped into distinct clusters using graph-based clustering. Finally, we utilized the Uniform Manifold Approximation and Projection (UMAP) to visualize the cell distribution. Based on well-recognized marker genes, we assigned the clusters to 6 major cell lineages (**Figure 1C**). Based on the expression levels of characteristic marker genes(**Supplementary Table S2**), we detected 6 major cell types previously reported, including endothelial cells (RAMP2), epithelial cells (KRT19, EPCAM), fibroblasts (COL1A2), mast cells (TPSAB1), macrophage cells (CD68), neutrophils (CSF3R) and T cells (CD3D), (**Figure 1D**). Comparing the cell proportions between the groups, we observed a higher proportion of neutrophils in the malignant group than in the non-malignant group (**Figure 1E**). Considering neutrophils serve as the first line of defense in innate immunity, the higher proportion in malignant group indicates that neutrophils respond to tumor development and exhibit increased infiltration in tumor tissues. We also compared the proportions of fibroblast, endothelial, epithelial cells, and immune cells between malignant and non-malignant groups (**Figure 1E**). Given the heterogeneity in cell components observed in TSCC ecosystems, more detailed investigation in molecular change is required to fully elucidate their complexity.

### Characterization of T-cell diversity and differentiation under malignant and non-malignant group

**Figure 2:**
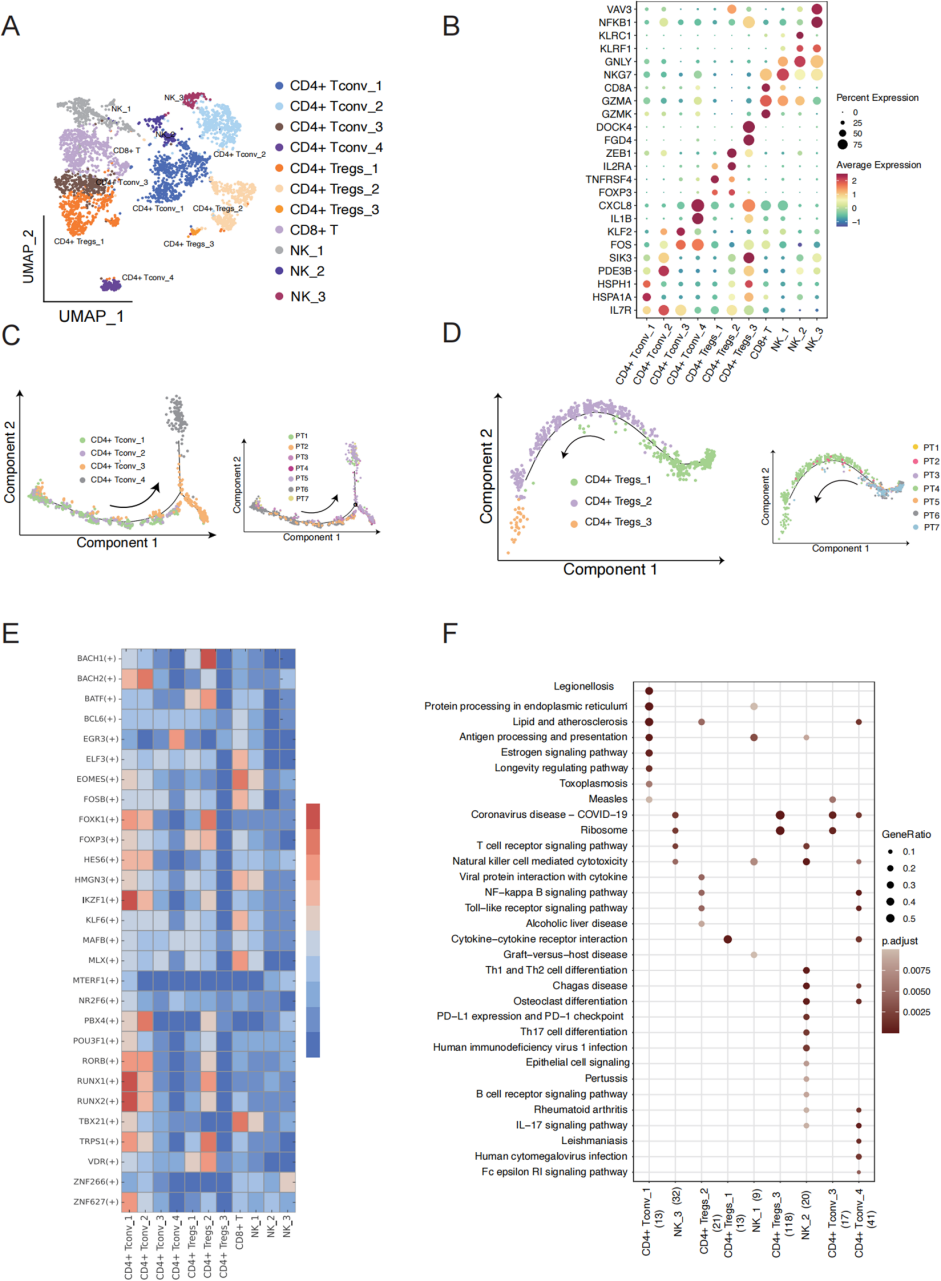
T cell subcluster characteristics and state transition in TSCC. **(A)** UMAP showing 11 clusters of 17,353 T cells colored by subpopulation. **(B)** Expression of the marker genes for the indicated cell subtypes. **(C)** Pseudotime trajectory of CD4^+^ T conv cell inferred by analysis with Monocle 2 (left) and cells of trajectory colored by sample (right). **(D)** Pseudotime trajectory of CD4^+^ T regs cell inferred by analysis with Monocle 2(left) and cells of trajectory colored by sample (right). **(E)** Heatmap showing the relative regulon activity in each T cell subtypes. The regulon activity is scored using RSS. **(F)** Comparison of pathway enrichment among different expression genes of T cell subtypes. X axis represents cell type and the number of DEG.

Using the expression levels of their respective marker genes, we identified and characterized the subpopulations of 3,466 T cells, including CD4^+^ Tconv, CD4^+^ Tregs, CD8^+^ T cells, and natural killer (NK) cells (**Figures 2A, B**) (**Supplementary Table S2**). While most T cell subpopulations were found in different patients, the relative abundance of each subpopulation greatly varied (**Supplementary Figure S2A**). For example, CD4^+^ Tconv_1 and CD4^+^ Tconv_2 was mostly found in non-malignant patients (PT6 and PT7), while more CD4^+^ Tconv_3 and CD4^+^ Tconv_4 was found in malignant patients (PT1-PT5). To explore the characteristics of the subpopulations of CD4^+^ Tconv, monocle trajectory analysis was performed to infer a differentiation trajectory. We found that the trajectory began with CD4^+^ Tconv_1, progressed to CD4^+^ Tconv_2, and finally to CD4^+^ Tconv_3 and CD4^+^ Tconv_4 (**Figure 2C**). Interestingly, the trajectory correlated with the patients, from samples with non-malignant (PT6 and PT7) to those with malignant. This disease-state association was further observed in CD4^+^ Tregs (**Figure 2D**), which the trajectory began with CD4^+^ Tregs_1, progressed to CD4^+^ Tregs_2, and finally to CD4^+^ Tconv_3 with matching clinical correlation. The conserved alignment between cellular trajectories and pathological states suggests potential functional reprogramming during malignant progression.

To better understand the key regulon that underlies the differentiation between the subpopulations of CD4^+^ Tconv and CD4^+^ Treg, we employed Single-Cell Regulatory Network Inference and Clustering (SCENIC) analysis to identify cluster-specific transcription factors (TFs) based on the gene expression of T cell subpopulations (**Figure 2E**). This analysis identified a set of TFs that provide critical insights into the molecular biology driving cellular heterogeneity within different T cell subtypes in TSCC (**Figure 2F**). Interestingly, CD4^+^ Tconv_1 and CD4^+^ Tconv_2 in non-malignant patients shared similar expression patterns compared to other cell types, while CD4^+^ Tconv_3 to CD4^+^ Tconv_4 in malignant patients also exhibited a similar expression of TF regulon compared to other cell types. This suggests that the differentiation between CD4 T cell subpopulations may be influenced by disease state. Notably, the expression of genes regulated by IKZF1 and RUNX1 was significantly upregulated in CD4^+^ Tconv_1. Previous studies have shown that IKZF1 plays an essential role in regulating the pathogenic program of CD4^+^ T cells (17, 18), while RUNX1 acts as a master regulator in various aspects of T cell immunity (19). In our pathway function enrichment analysis, we observed a transition in cell type function along the cell trajectory. The marker genes of CD4^+^ Tconv_1 cell are enriched in KEGG pathways and GO terms related to protein processing, protein folding, and ATP hydrolysis (**Figure 2F**, **Supplementary Figure S2B**). Notably, the marker genes functions of CD4^+^ Tconv_4 enriched in IL−17 signaling pathway, IgG binding, cytokine receptor binding and immunoglobulin binding, which play a crucial role in reshaping the immune microenvironment of TSCC. Our analysis also revealed a progressive decline in immune function from CD4+ Treg_1, CD4+ Treg_2 to CD4+ Treg_3. CD4+ Treg_1 cell exhibited elevated activity in cytokine-cytokine receptor interactions and cytokine activity, while CD4+ Treg_2 cells demonstrated heightened activity in the NF-kappa B signaling pathway and chemokine receptor binding. In contrast, CD4+ Treg_3 cells were primarily associated with non-immune functions, including rRNA binding and involvement in the ribosome pathway. Natural Killer cells (NK cells) are also essential components of the cancer microenvironment and have shown increasing promise in tumor (20, 21). We found that the marker genes of NK_2 are enriched with KEGG pathways associated with cancer defense, including PD-L1 expression, PD-1 checkpoint pathway in cancer, T cell receptor signaling pathway, and IL-17 signaling pathway, as well as GO terms such as immune receptor activity and MHC protein complex binding (**Figure 2F**, **Supplementary Figure S2B**). These findings identify candidate regulators that may contribute to the cell state transition between malignant and non- malignant patients.

### Gene expression change between malignant and non-malignant group of fibroblast and neutrophils cells

**Figure 3:**
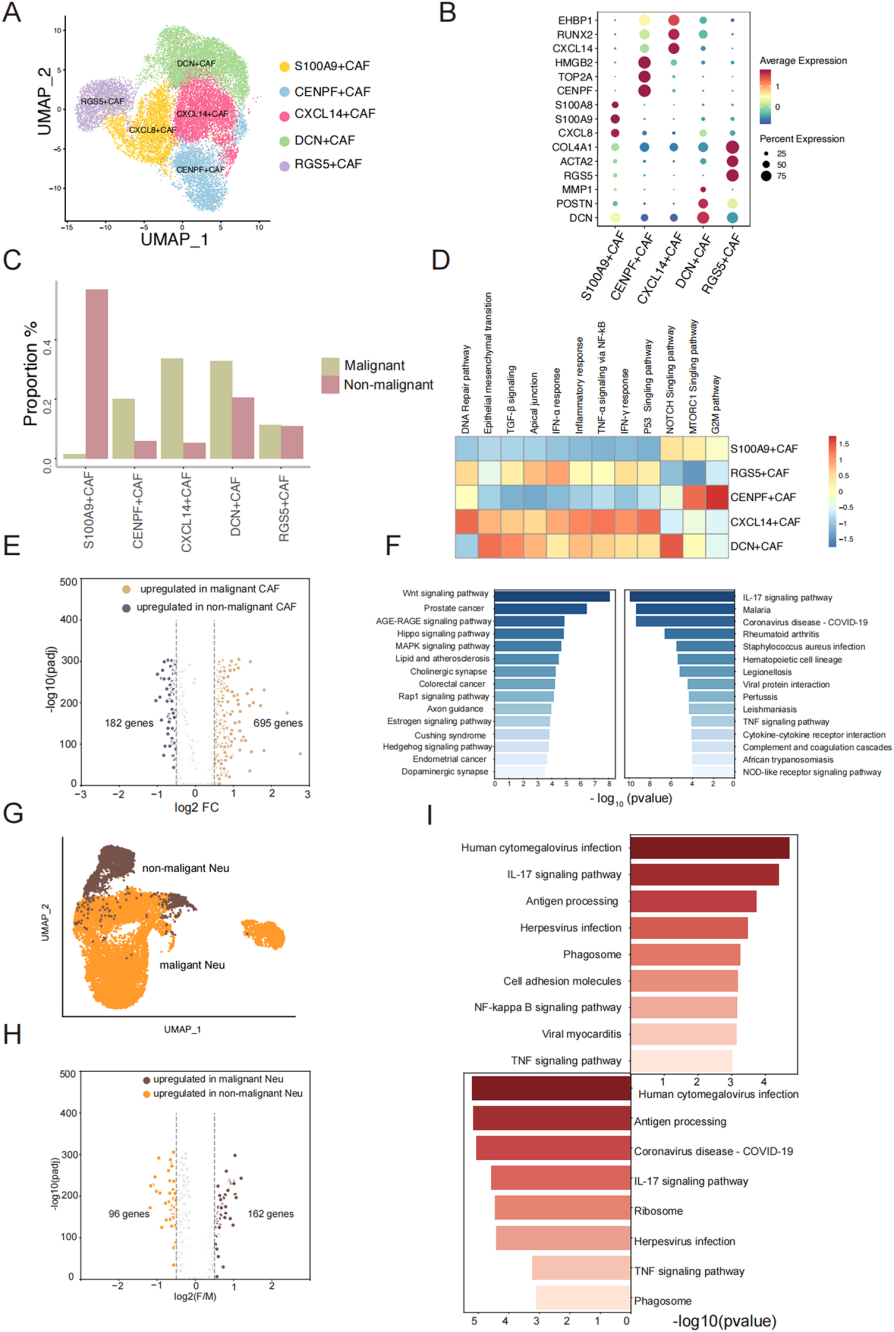
Gene expression change of fibroblast and neutrophils between malignant and non-malignant. **(A)** UMAP plot of fibroblast cells and colored by subtype. **(B)** The dotplot represents the marker expression of different fibroblast subtypes. **(C)** The proration of five fibroblast subtypes between two groups. **(D)** Enrichment pathway of five fibroblast subtypes. **(E)** Volcano plot showing differential expressed genes between malignant fibroblast cells and non-malignant fibroblast cells. **(F)** Pathway enrichment analysis of significantly upregulated genes (left: malignant sample, right: non-malignant sample). **(G)** UMAP plot of Neutrophil cells and colored by sample type. **(H)** Volcano plot showing differential expressed genes between malignant Neutrophil cells and non-malignant Neutrophil cells. **(I)** Top enrichment Pathway of significantly upregulated genes of malignant Neutrophil cells and non-malignant Neutrophil cells (left: malignant sample, right: non-malignant sample).

Fibroblasts are the predominant cells found in both non-malignant and malignant TSCC groups. They play multiple roles in tumorigenesis, cancer development, and resistance to various therapeutic strategies within the tumor microenvironment (TME) (22, 23). We further conducted UMAP analyses to identify the cell populations within the fibroblast cluster (**Figure 3A**). We identified five transcriptionally distinct subpopulations from a cohort of 17,495 fibroblast cells and these subpopulations were assigned specific cell type names based on the overlap between representative marker genes and the top marker genes characteristic of each fibroblast subtype (**Figure 3B**) (**Supplementary Table S2**). Obviously, we found a significantly higher proportion of S100A9+ CAF in the non-malignant group, while the proportion of CXCL14+ CAF was notably elevated in the malignant group (**Figure 3C**). To better understand the biological characteristic among different subgroups, we applied ssGSEA to perform the functional enrichment analysis for comparing different fibroblast subclusters. Epithelial mesenchymal transition, TGF-β signaling pathways and P53 singling pathway were found to be up-regulated in CXCL14+CAF, which the cell type proportion was prominently in malignant group. The characteristics of DCN+CAF were found to be similar to CXCL14+CAF in terms of cell type proportion and enrichment pathway (**Figure 3D**). In addition, CENPF+CAF demonstrated a significantly higher cell type proportion in the malignant group and exhibited a high enrichment score for the G2M pathway (**Figure 3D**). We also observed the expression level of p53 pathway can be a hallmark to distinguishes non-malignant and malignant TSCC groups.

To understand the molecular changes after a benign tumor deteriorates into a malignant tumor, we performed differential expression analysis between 5124 fibroblast cells in non-malignant and 13120 fibroblast cells in malignant group (**Figure 3E**). We identified 695 genes that were upregulated in malignant fibroblast cells and 182 genes that were upregulated in non-malignant fibroblast cells (**Figure 3F**) (**Supplementary Table S3**). This indicates that the genes are more highly expressed in malignant fibroblast cells. We observed elevated expression levels of CXCL14 and SPP1 in malignant samples compared to non-malignant samples with consistent patterns observed across both the scRNA-seq data and the TCGA-LUSC dataset (**Supplementary Figures S3A-D**). To describe the functions of highly expressed genes in non-malignant and malignant fibroblast cells, respectively, KEGG pathway analysis revealed that genes upregulated in malignant fibroblast cells were highly associated with pathways including Wnt signaling pathway, AGE-RAGE signaling pathway, Hippo signaling pathway, MAPK signaling pathway, and Rap1 signaling pathway, etc. (**Figure 3F**). Previous studies have shown that proteins or molecular factors produced by these pathways can promote tumor aggressiveness (24–26). The upregulated DEGs in non-malignant fibroblast cells were enriched in the IL-17 signaling pathway and TNF signaling pathway (**Figure 3F**). IL-17 promotes an anti-tumor cytotoxic T cell response to enhance the antitumor effects, and TNF can stimulate the proliferation of other immune cells to limit tumor progression (27, 28). We observed an increase in neutrophil cell proliferation from 11.07% in non-malignant to 29.25% in malignant groups (**Figure 1E**), which led us to hypothesize that changes in cell phenotype might imply biological variation in the two groups. We conducted a similar analysis as above and identified 163 genes with significantly higher expression in malignant samples and 96 genes with significantly higher expression in non-malignant samples (**Figure 3G, H**) (**Supplementary Table S4**). Pathway analysis of these upregulated genes showed a similar pathway enrichment profile between non-malignant and malignant neutrophil cells, including the IL-17 signaling pathway and TNF signaling pathway (**Figure 3I**). As primary responders in the innate immune system, neutrophils display conserved activation patterns when exposed to similar inflammatory microenvironments, may induce similar molecular adaptations in neutrophils. One different pathway was the NF-kappa B signaling pathway, which is an active player in human cancer (29). Overall, our observations revealed a greater number of upregulated genes in fibroblast cells and neutrophil cells in malignant group compared to the non-malignant group. We believe that the elevated expression profile in the malignant group can be attributed to the activation of relating pathways with tumor cell proliferation and growth.

### Unique molecular characteristics of TSCC epithelial cells

**Figure 4:**
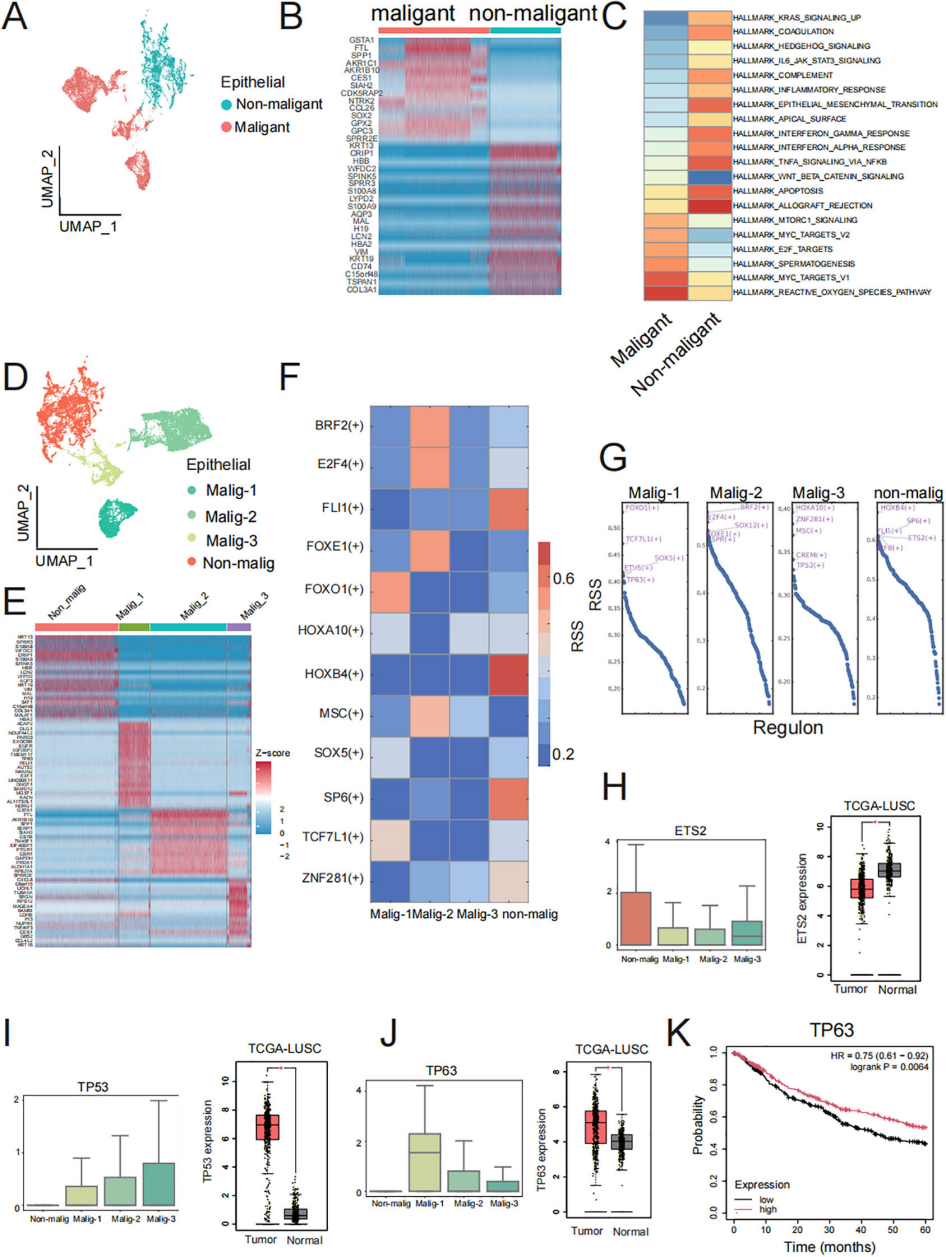
Malignant and non-malignant heterogeneity of cancer cells. **(A)** UMAP visualization of malignant and non-malignant epithelial cell groups. **(B)** Top 30 DEGs in malignant and non-malignant epithelial cell groups. **(C)** Heatmap of GSVA of the 50 hallmark gene sets in the MSigDB database between Malignant and non-malignant epithelial cells. **(D)** UMAP visualization of non-malignant and three malignant epithelial cell subclusters. **(E)** Top 30 DEGs in non-malignant and three malignant subclusters cancer cell. **(F)** Specific TF regulons identified in non-malignant and three malignant subclusters. Color key from blue to red represents the RSS scores from low to high. **(G)** Transcription factors were sorted by RSS value in non-malignant and three malignant epithelial cell subclusters. Top five transcription factor of each subclusters were labeled by red font. **(H, J)** Boxplot of ETS2, TP63 and TP53 gene expression in non-malignant and three malignant epithelial cell subclusters and boxplot of ETS2, TP63 and TP53 gene expression in TCGA-LUSC dataset. The three genes were specific transcription factors in non-malignant, malig-1, and malig-3 respectively, along with DEGs in corresponding clusters. **(K)** Prognostic analysis of TP63 expression levels (high vs low) on overall survival in TCGA-LUSC patients.

We conducted differential expression analyses to identify specifically expressed genes in TSCC malignant epithelial cells compared to non-malignant epithelial cells, which were visualized by UMAP analysis (**Figure 4A**). After differential analysis, we identified a total of 276 DEGs, of which 110 were upregulated in malignant epithelial cells and 166 were upregulated in non-malignant epithelial cells. The top 30 DEGs in each group were presented in a heatmap (**Figure 4B**). These top DEGs represent the genes driving the transfer of non-malignant epithelial cells to malignant epithelial cells in molecular biological behavior. Subsequently, we used GSVA to investigate the functions of DEGs in the two groups of genes (**Figure 4C**). The non-malignant epithelial cell-specific genes were enriched in signaling pathways related to the inflammatory, such as the inflammatory response and interferon-gamma response. Malignant epithelial cells were specifically enriched in signaling pathways related to v-Myc targets, mTORC1 singling and the reactive oxygen species pathway. These results indicate that these DEGs contribute to the phenotypic differences between non-malignant epithelial and malignant epithelial cells. To describe the biological differences at the molecular level in more detail, we attempted to further subdivide the malignant epithelial cells and identified four related malignant epithelial cell subclusters and one related non-malignant epithelial cell cluster, visualized by UMAP analysis (**Figure 4D**). We found 232 DEGs in non-malignant epithelial cells and 63, 45, 30 DEGs in the four malignant epithelial cell groups, respectively, and the top 30 DEGs in each group were presented in a heatmap (**Figure 4E**). We hypothesized that DEGs in these subsets may be the major drivers of the transformation from benign to malignant epithelial cells. We ran pySCENIC to identify specific transcription factor activities from the gene expression of their targets (TF regulon) between subclusters (**Figure 4F**). We found that TP63 in malig_1, TP53 in malig_3, and ETS2 in the non-malignant group were core transcription factors that regulate a vast array of downstream genes (**Figure 4G**). Interestingly, ETS2, TP53, and TP63 were also DEGs in non-malignant and malignant epithelial cells. The expression trends of those genes show concordance between our single-cell data and TCGA bulk RNA-seq results (**Figures 4H-J**). ETS2 is essential for cell signaling pathways involved in the cellular response to growth factors and may contribute to benign TSCC proliferation (30, 31). The high expression of TP53 and TP63 as specific transcription factors regulate the expression of downstream genes by regulation network and influence cell growth and the formation of tumors (32, 33). The expression of TP63 demonstrated significant prognostic value, with elevated levels of expression being associated with enhanced overall survival (**Figure 4K**). In summary, our results indicate that these DEGs, serving as specific transcription factors in subsets, are key genes for the difference between non-malignant and malignant epithelial cells and may improve the prognosis of TSCC.

### Specific cell-cell communication between malignant and non-malignant group

Next, using CellChat (34), we identified 1210 ligand-receptor pairs interactions in all ten major cell types (**Supplementary Figure S3A**). The TME is shaped by complex cell-cell interactions between immune cells, stromal cells, and tumor cells that ultimately modulate tumor growth, leading to tumor deterioration and metastasis.

**Figure 5:**
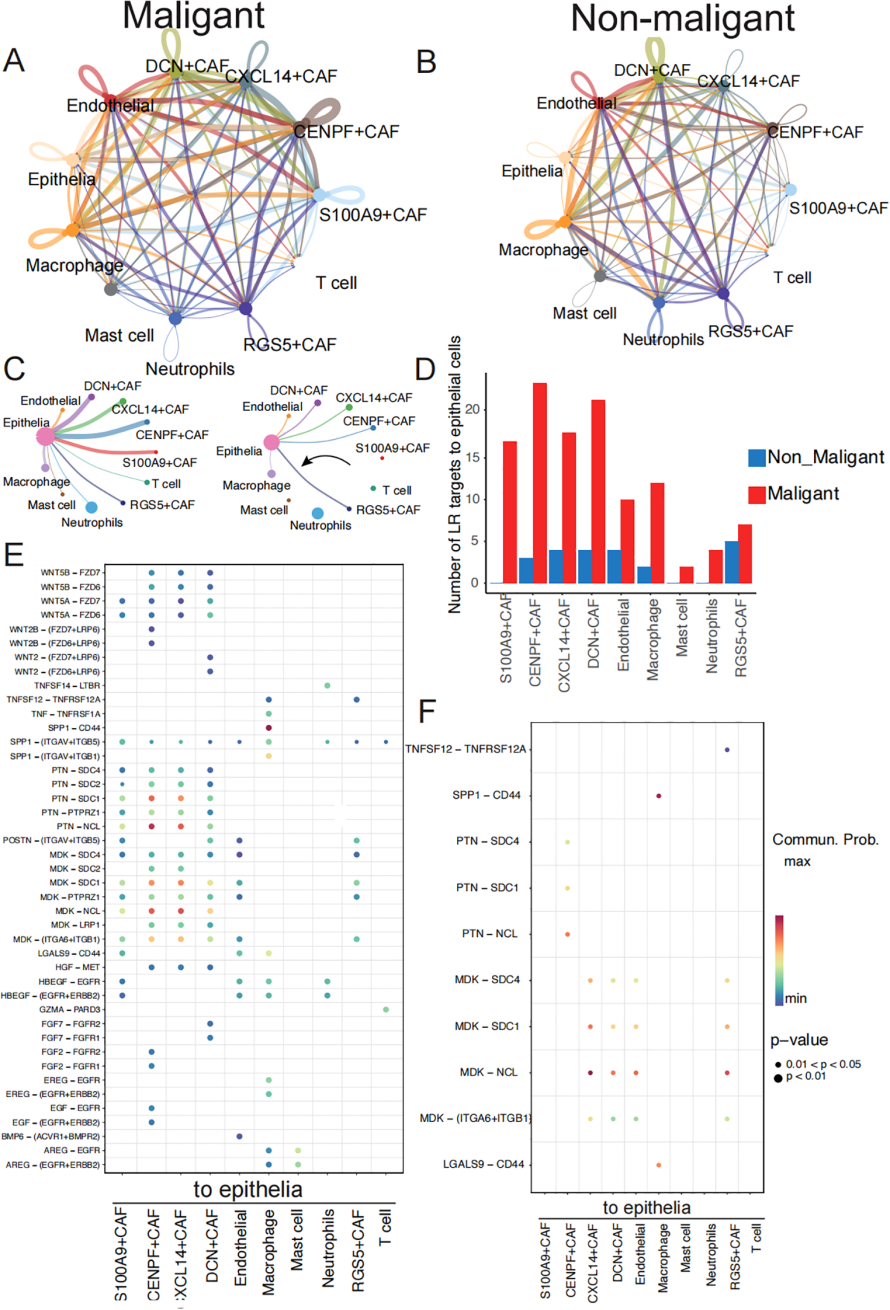
Communication analysis exhibits malignant-specific ligand–receptor interactions. **(A)** Communication between cells within a major cell type in malignant samples colored by cell subtypes. Two cell populations are represented by the size of their flow, which represents the number of ligand-receptor pairs. **(B)** Cell-cell communications within major cell type in non-malignant samples. **(C)** Detailed view of the ligands-receptor pairs sender from stromal cells and immune cells to epithelia in two groups. The width of line represents the number of ligands-receptor pairs. **(D)** The number of ligand-receptors pairs from stromal cells and immune cells to epithelia cells between malignant and non-malignant samples. **(E)** The dot plot generated by CellChat demonstrates potential ligand-receptor pairs in malignant group. The colored dots represent communication probability, while the size of the dots represents the P Value. **(F)** The dot plot generated by CellChat demonstrates potential ligand-receptor pairs in non-malignant group.

Previous studies have reported on the interplay among immune cells, stromal cells, and tumor cells in the tumor microenvironment (14, 35, 36). To explore the specific molecular pairs mediating cell-cell interactions between non-malignant and malignant cells in these cell types, which may drive benign neoplasms into malignant neoplasms, we performed cell-cell interaction analysis in non-malignant and malignant cells separately (**Figures 5A, B**). Our interest focus on the interaction from stromal cells and immune cells to epithelia cells. We considered the biological different of epithelia between two groups may be the different cell-cell interaction from other cells to epithelia cells. We identified a higher number of ligand-receptor pair interactions in the malignant groups compared to the non-malignant group, with 134 interactions observed in the former and 26 interactions in the latter (**Supplementary Table S5**). We found the ligand-receptor interaction number from DCN+CAF, CXCL14+CAF, CENPF+CAF, S100A9+CAF and RGS5+CAF was increased in malignant group (**Figures 5B, C**). Besides, 90 ligand-receptor pairs specifically acting on epithelia cells in the malignant group (**Supplementary Table S6**).

These pairs involve EGF, WNT, SPP1 and HGF signaling pathways (**Figures 5C, D**) (**Supplementary Table S7**). Specifically, the HGF signaling pathway was observed to have a significant effect on the interaction between fibroblasts and malignant tumor cells (**Supplementary Figure S3B**). The HGF ligand gene was expressed at high levels in the malignant group of fibroblast cells relative to the non-malignant group (**Supplementary Figure S4C**). This signaling pathway plays a vital role in promoting cancer motility and invasion by remodeling and reorganizing the cytoskeleton (37, 38). To determine the clinical significance of HGF expression, we found HGF in the genes list whose expression levels have been correlated with survival outcomes in LUSC patients (**Supplementary Table S8**) and then we conducted survival analyses of HGF expression using Cox proportional hazards regression visualized as a Kaplan-Meier plot (39). Patients with higher expression of HGF secreted by fibroblast cells had significantly improved overall survival compared to those with lower expression of HGF (P = 0.0012) (**Supplementary Figure S4D**). We also observed specific interaction between fibroblasts and malignant tumor cells through the interaction of WNT5B and WNT2B with its downstream receptor in the WNT signaling pathway (**Figures 5E, F**), which has been linked to supporting tumor cell proliferation, metabolism, and metastasis (40–43). Furthermore, the malignant group exhibited a higher transmission of ligands AREG and MDK from immune or fibroblast cells to epithelial cells compared to the non-malignant group. Elevated AREG expression was significantly correlated with improved overall survival (p = 0.0019) (**Supplementary Figure S4E**), while reduced MDK expression was associated with a more favorable prognosis (p = 0.046) (**Supplementary Figure S4F**).

As a result, we speculate that malignant-specific ligand-receptor pairs play a greater role in tumor progression, and their expression level is significantly associated with the overall survival ratio of patients with TSCC. There is a possibility that these pairs may become critical targets in cancer therapy.

## Discussion

In this study, we present a valuable comprehensive analysis of cell types in malignant and non-malignant trachea squamous cell carcinoma using scRNA-seq. We identified 10 major cell types from TSCC, providing a valuable resource for future investigations of cellular diversity and components of cancer microenvironments. We also found that some subtypes of T cells and neutrophils undergo cell state transformation between the early and later stages of chemotherapy. Furthermore, we focused on the molecular differences and group-specific cell-cell interactions between malignant and non-malignant TSCC, suggesting cancer-specific transcriptional regulation and expression.

From the cellular composition analysis, we showed a higher content of neutrophils in malignant samples (25.02%) than in non-malignant samples (11.07%). Previous literature has demonstrated that tumor cells produce granulocyte colony-stimulating factor (G-CSF) (44), which may increase the release of neutrophils in the bone marrow, leading to an increase in neutrophils in the TSCC microenvironment. As a result of environmental changes, CD4 T cells are able to eliminate tumor cells in various ways and exhibit a high degree of plasticity and differentiation potential (45–47). It is vital for the immune response to be shaped by regulatory B cells (Tregs) and conventional T cells (Tcon), which also regulate the body’s tolerance to infection, making them crucial in the regulation of tumor immunity (48, 49). We observed a trend of transitions from no chemotherapy type to chemotherapy type in CD4 Tregs and CD4^+^ TCons by trajectory analysis, which may be due to chemotherapy perturbing gene regulatory and metabolic networks, leading to differentiation to adapt to external simulation. In terms of gene regulatory and metabolic networks, subtype-specific biological processes detected by pathway enrichment and regulons identified by SCENIC analysis may play a crucial role in cell subpopulation differentiation.

Differential gene expression analysis between cells of the same type in malignant and non-malignant microenvironments can help uncover driver genes leading to tumor deterioration and avoid differences due to cell types. We observed more upregulated genes in the malignant group compared to the non-malignant group, suggesting that cancer metabolism is more active and requires increased nutrient uptake to provide energy for its rapid development and growth (50–52). Interestingly, we also found enrichment of IL-17 signaling pathway, AGE-RAGE signaling pathway, and TNF signaling pathway in macrophage cells between malignant and non-malignant groups (**Supplementary Figure S5A**), indicating the crucial role of these pathways in antitumor immune response (52). We also found some specific transcriptional regulons that are differentially expressed between malignant subsets and non-malignant groups, which may play a more critical role in exploring phenotypic differences.

A tumor’s microenvironment consists of immune cells, tumor cells, and the stroma surrounding them, which has a comprehensive cell-cell communication effect on tumor cells and is an important cause of continuous tumor growth and metastasis. Immune cells trigger downstream signaling through cognate receptors, altering transcription factor activity and gene expression, leading to abnormal biological processes in tumors. In our study, we identified 1210 ligand-receptor pairs of interactions among ten cell types in all samples. In the malignant group, we observed a higher presence of ligand-receptor interaction pairs, indicating that the adaptive immunity stimulated by the tumor microenvironment can potentially promote or suppress tumor growth. Previous studies have reported that the adaptive immune response of some immune cells is specifically triggered by antigenic proteins expressed in tumors (53–55). Comparing cell-cell communication in malignant and non-malignant samples, a set of ligand-receptor pairs shows group-specific interaction. We believe that those tumor-specific ligand-receptor pairs may play a major role in the increase of tumor deterioration and metastasis compared to the malignant group.

This study had some notable limitations. Firstly, due to the scarcity of malignant and non-malignant samples, the number of samples collected was not balanced enough. To address this, we validated the expression patterns of key genes identified in our single-cell RNA sequencing (scRNA-seq) analysis by comparing malignant and non-malignant groups using the TCGA-LUSC dataset. And critical pathways such as cell cycle regulation, cell-cell junction integrity, extracellular matrix remodeling, and epithelial cell proliferation exhibited similar activation patterns of tumor-associated highly expressed genes in both the scRNA-seq and TCGA-LUSC datasets (**Supplementary Figures S5B, C**). Secondly, the chemotherapy information of our samples was complex, including different drug therapies and treatment times, so we used related information to divide samples into chemotherapy and non-chemotherapy groups. The cell state transition of immune cells after chemotherapy requires further study. Lastly, separate sampling locations and physiological variability may introduce heterogeneity into our analysis. This underscores the need for future studies involved in larger sample and multi-omics data to precisely characterize the molecular transitions during TSCC development.

In summary, the single-cell data from TSCC provides a vital and unique insight into the characterization of stromal and immune landscape in the TSCC microenvironment and identifies malignant-related genes and ligand-receptor pairs. It is necessary to carry out follow-up mechanistic studies in order to determine the role of group-specific genes and ligand-receptor pairs in promoting tumor deterioration and metastasis.

## Data Availability

The datasets presented in this study can be found in online repositories. The names of the repository/repositories and accession number(s) can be found in the article/**Supplementary Material**.

## Supplementary material

Supplementary Figure 1
**(A)** Violin plots show gene expression number, UMI counts and proration of mitochondrial after quality control. **(B)** UMAP plot of all cells, colored by patients.

Supplementary Figure 2
**(A)** T subpopulations proration in seven patients. **(B)** GO pathway enrichment in T subpopulations. X axis represents subpopulation and its marker genes number.

Supplementary Figure 3
**(A, B)** The expression of CXCL14 in fibroblast cells between malignant and non-malignant groups in scRNA-seq and between tumor and normal groups in TCGA-LUSC dataset. **(C, D)** The expression of SPP1 in fibroblast cells between malignant and non-malignant groups in scRNA-seq and between tumor and normal groups in TCGA-LUSC dataset.

Supplementary Figure 4
**(A)** The cell-cell communications within major cell type in all samples colored by cell subtypes. **(B)** Visualize HGF signaling pathway by Chord diagram between major cell types in malignant samples(left). Gene expression level of HGF genes between malignant and non- malignant group(right). **(D)** Overall survival curves for patients in TCGA-LUSC datasets according to the high and low expression levels of HGF. **(E, F)** Overall survival analysis for patients in TCGA-LUSC datasets according to the high and low expression levels of AREG and MDK.

Supplementary Figure 5
**(A)** KEGG pathway enrichment analysis of significantly upregulated genes in macrophage of malignant group in TSCC. **(B)** Pathway enrichment analysis of significantly upregulated genes in all cells from malignant groups than non-malignant in scRNA-seq data. **(C)** Pathway enrichment analysis of significantly upregulated genes in malignant groups in TCGA-LUSC.

Supplementary Table 1 Clinical information of seven patients.

Supplementary Table 2 Top20 marker genes used to annotate major cell types and subtypes.

Supplementary Table 3 Upregulated genes calculated between malignant group fibroblasts and non-malignant group fibroblasts cell.

Supplementary Table 4 Upregulated genes calculated between malignant group neutrophils and non-malignant group neutrophils cell.

Supplementary Table 5 Significantly ligand-receptor pairs from stromal and immune cells to epithelia cells in malignant and non-malignant group.

Supplementary Table 6 Ligand-receptor pairs from stromal and immune cells in malignant group.

Supplementary Table 7 Ligand-receptor pairs from stromal and immune cells in non-malignant group.

Supplementary Table 8 All genes list whose expression levels correlated with survival outcomes in TCGS-LUSC patients.
