# Supplementary figures and images for: Comprehensive single-cell sequencing reveals the tumor microenvironment and tumor-specific characteristics in trachea squamous cell carcinoma
Source: Front Oncol. 2025 Aug 12;15:1575647. doi: 10.3389/fonc.2025.1575647 (PMC12378060; doi:10.3389/fonc.2025.1575647)

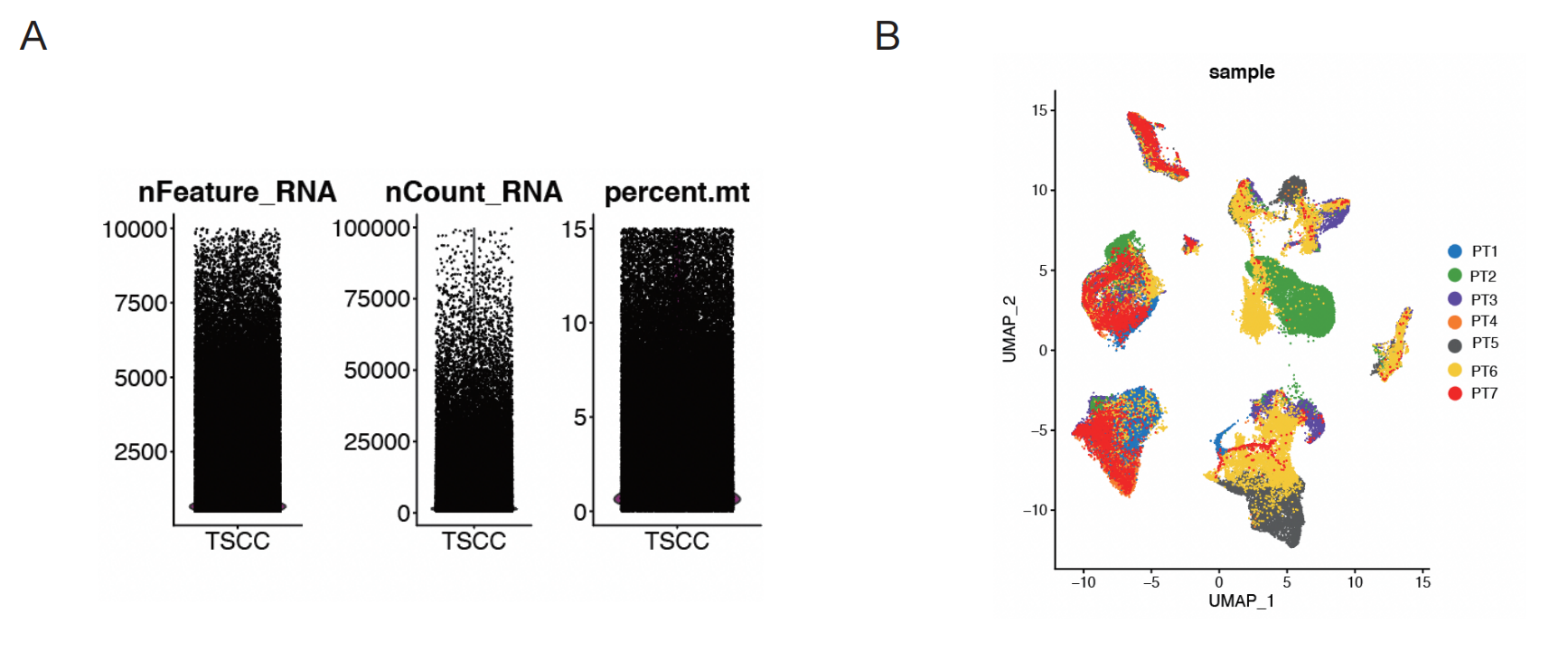

Supplement: Supplementary Figure 1 — (A) Violin plots show gene expression number, UMI counts and proration of mitochondrial after quality control. (B) UMAP plot of all cells, colored by patients. [file DataSheet1.zip › Supplementary file 1.TIF]

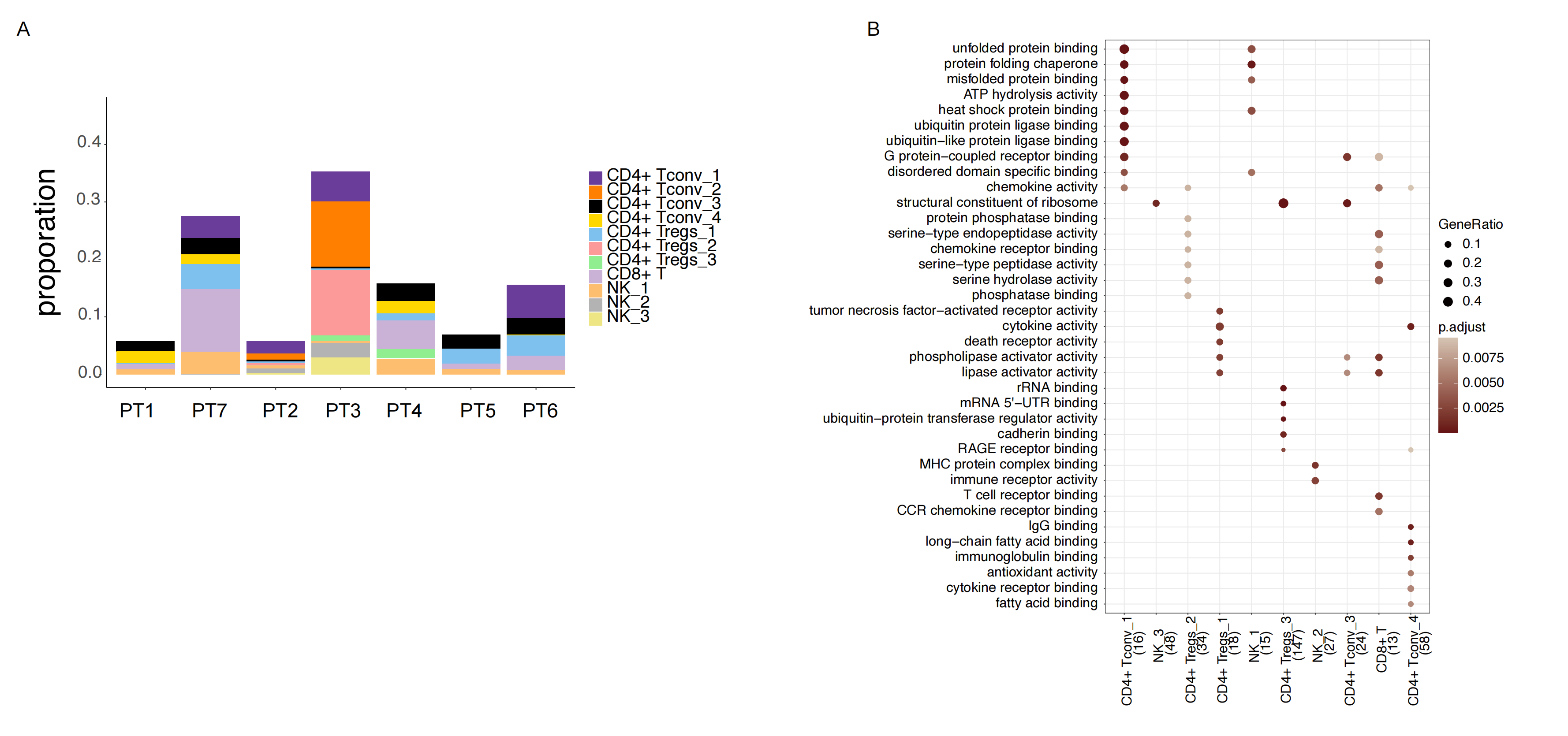

Supplement: Supplementary Figure 1 — (A) Violin plots show gene expression number, UMI counts and proration of mitochondrial after quality control. (B) UMAP plot of all cells, colored by patients. [file DataSheet1.zip › Supplementary file 2.TIF]

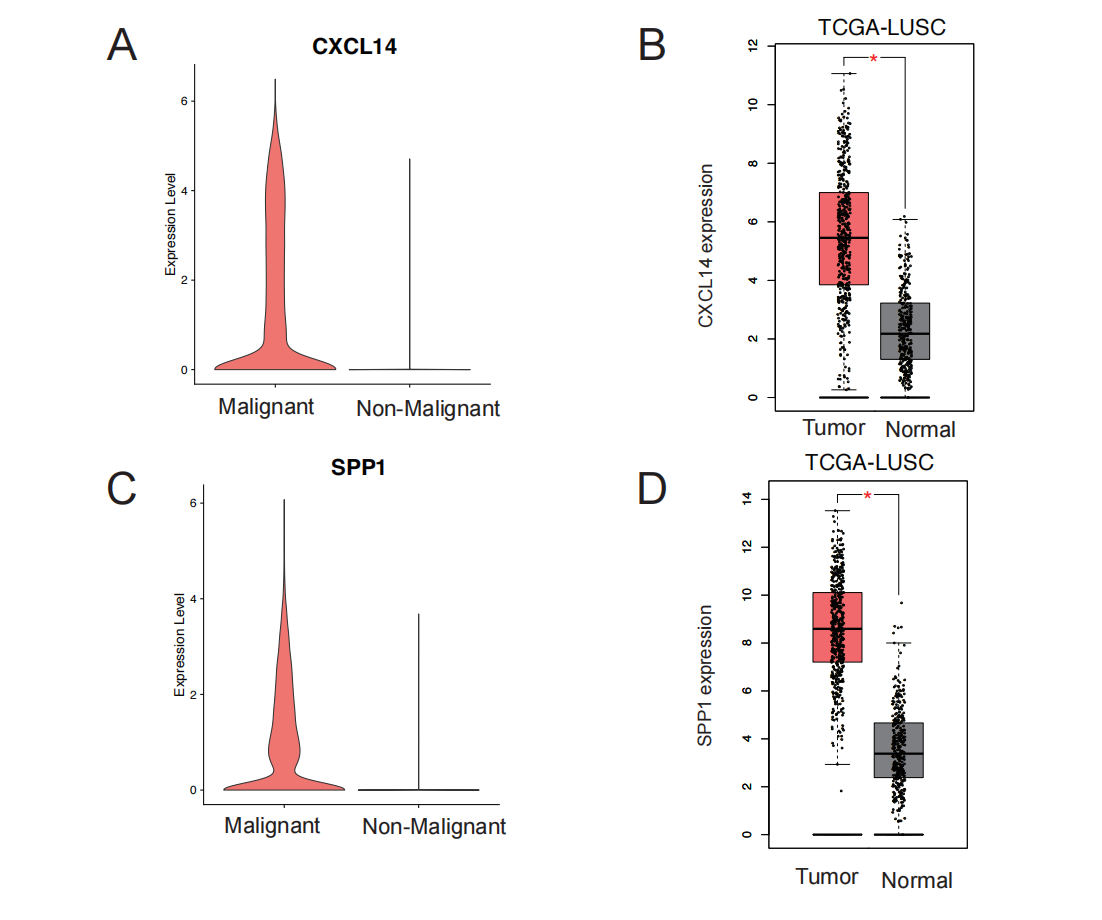

Supplement: Supplementary Figure 1 — (A) Violin plots show gene expression number, UMI counts and proration of mitochondrial after quality control. (B) UMAP plot of all cells, colored by patients. [file DataSheet1.zip › Supplementary file 3.TIF]

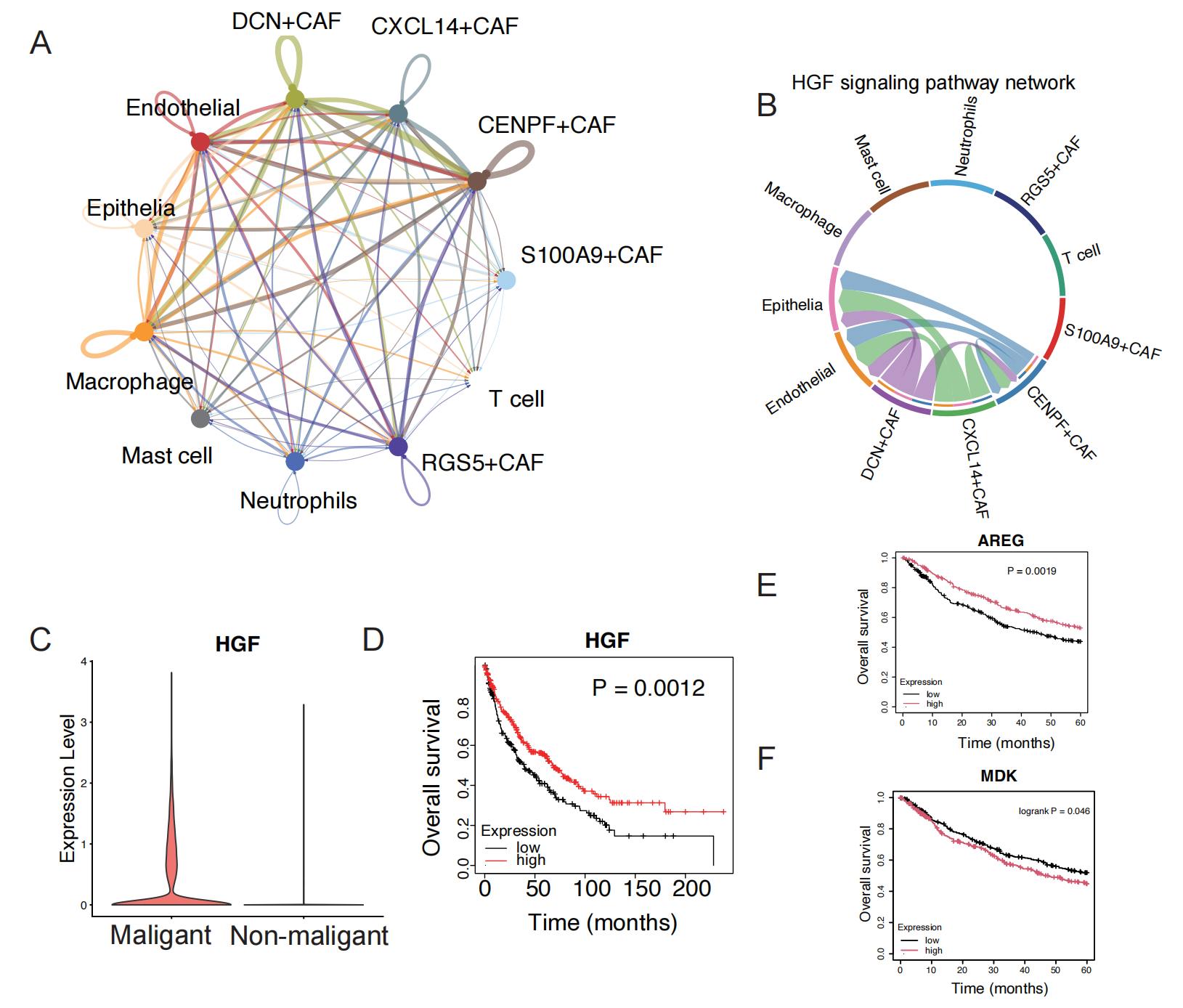

Supplement: Supplementary Figure 1 — (A) Violin plots show gene expression number, UMI counts and proration of mitochondrial after quality control. (B) UMAP plot of all cells, colored by patients. [file DataSheet1.zip › Supplementary file 4.TIF]

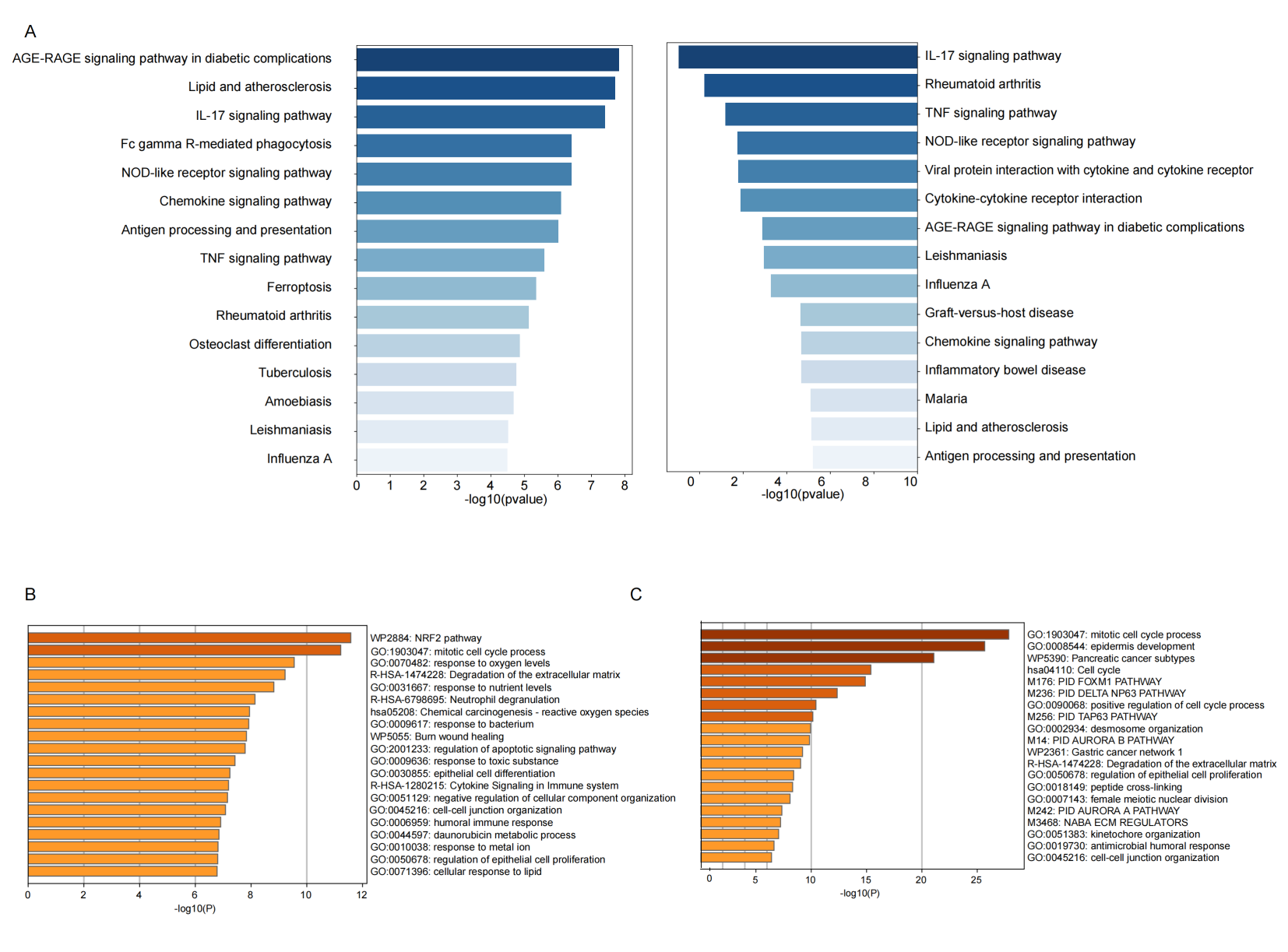

Supplement: Supplementary Figure 1 — (A) Violin plots show gene expression number, UMI counts and proration of mitochondrial after quality control. (B) UMAP plot of all cells, colored by patients. [file DataSheet1.zip › Supplementary file 5.TIFF]
